# Antenatal diagnosis of extralobar pulmonar sequestration

**DOI:** 10.11604/pamj.2014.19.54.4698

**Published:** 2014-09-23

**Authors:** El Mhabrech Houda, Zrig Ahmed, Ksia Amine, Ben Salem Amina, Faleh Raja, Hafsa Chiraz

**Affiliations:** 1 Department of Radiology B, Maternal and Fetal Unit, Fattouma Bourguiba Hospital-Monastir, Tunisia; 2 Department of Radiology A, Fattouma Bourguiba Hospital-Monastir, Tunisia; 3 Department of Pediatric Surgery, Fattouma Bourguiba Hospital-Monastir, Tunisia; 4 Department of Gynecology and Obstetrics, Maternal and Fetal Unit, Fattouma Bourguiba Hospital-Monastir, Tunisia

**Keywords:** Extralobar pulmonary sequestration, antenatal diagnosis, ultrasonography, MRI

## Abstract

Extralobar pulmonary sequestrations (ELS) are masses of non-functioning lung tissue that are supplied by an anomalous systemic artery and do not have a bronchial connection to the native tracheobronchial tree. On prenatal ultrasonography, an ELS appears as a well-defined echodense, homogeneous mass. Detection by color flow Doppler ultrasonography of a systemic artery from the aorta to the fetal lung lesion is a pathognomonic feature of fetal ELS. MR imaging may help in the diagnosis of pulmonary sequestration by demonstrating a solid, well-defined mass, and the feeding artery. In this case report, we describe the sonographic and MR diagnosis of an ELS in a fetus at 22 weeks gestation with a review of the available literature.

## Introduction

Pulmonary sequestration is an embryonic mass of lung tissue that has no identifiable bronchial communication and that receives its blood supply from 1 or more anomalous systemic arteries. Multiple feeding vessels may be present. This congenital anomaly can be classified as extralobar sequestration (ELS; 25% of patients with pulmonary sequestration) or intralobar sequestration (ILS; 75% of patients with pulmonary sequestration). The aim of this article is to report one case of extra-lobar pulmonary sequestration, which was diagnosed in utero by US and MRI with focus on the imaging characteristics of this rare anomaly.

## Patient and observation

**Figure 1:**
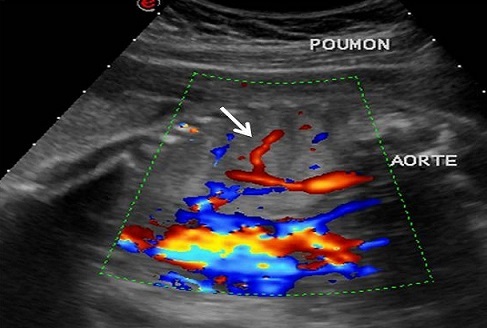
Left sagittal sonogram of fetal chest: echogenic pulmonary mass and visualisation of systemic feeding artery arising from the abdominal aorta (arraw)

A 28 year-old primipara has been referred by her obstetician to the radiology department to undergo fetal MRI because hyperechoic mass in the left lung had been detected on an ultrasound examination performed at 22 weeks of amenorrhea. A repeat ultrasonography showed a hyperechoic mass in the left lung and the combination of color Doppler ultrasound allowed visualization of a vessel arising from the descending aorta, which supplied the mass (Figure 1). The diaphragm was of normal appearance on US. The diagnosis of extralobar pulmonary sequestration was made.

**Figure 2:**
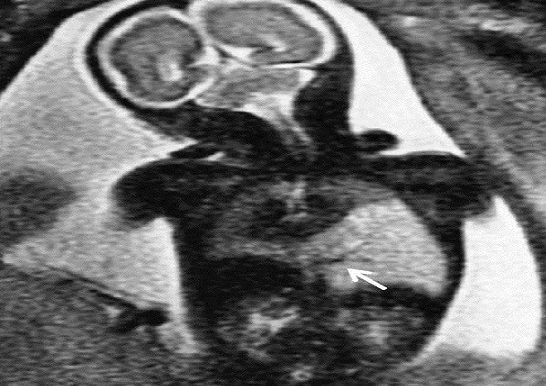
Coronal prenatal MR image: left-sided triangular pulmonary mass of increased signal intensity. Note systemic feeding artery arising from the abdominal aorta (arraw)

Magnetic resonance imaging was also performed using a 1.5 Tesla magnetic field, with multiplanar T2 single-shot turbo spin echo sequences. It revealed a well-defined triangular mass with homogeneous high-signal intensity when compared with normal lung tissue in the left down lung field, which was compatible with pulmonary sequestration (Figure 2). MRI did not show any diaphragm or other thoracic abnormality.

A male newbon was spontaneously delivered at 38 weeks and was asymptomatic at birth. Postnatal CT scan shows a homogeneous mass in the posterior segment of the left lower lobe. The infant underwent surgery at 12 months because of persistence of the masse and intraoperative photograph obtained shows ectopic masse inferior to the lung with a vascular supply from the descending aorta.

## Discussion

Pulmonary sequestration refers to aberrant formation of segmental lung tissue that has no connection with the bronchial tree. It is a bronchopulmonary foregut malformation. The estimated incidence is at 0.1% [1–3]. Extralobar sequestration (ELS) is less common (15 - 25% of all sequestrations) and recogonised male predilection (M:F ratio = 4/1) [1–3]. It is usually present in the neonatal period with respiratory distress, cyanosis and / or infection. The sequestrated lobe is usually intra-thoracic, rarely intraabdominal (10% of cases) [1–3]. Bilateral forms have also been described [3]. The extralobar form has its own pleural investment and systemic venouse drainage [2, 4]. In ELS, 80% of sequestrations lie between the lower lobe and the diaphragm, as found in our case [2]. Lesions are usually located in the region of the posterior basal segments of the lower lobes. Left-sided lesions are more common than right-sided lesions [4]. The mass may be closely associated with the esophagus, and fistulae may develop. Subdiaphragmatic ELS lesions can mimic masses arising in various organs, such as the adrenal gland.

In addition, ELS frequently is associated with other congenital extrapulmonary anomalies. It has a systemic arterial supply, usually from the thoracic or abdominal aorta [1–4].

Occasionally, the systemic arterial supply originates from the celiac or splenic artery or from the intercostal, subclavian, or even coronary arteries [1, 3]. Venous drainage occurs via the systemic circulation. ELS can be associated in 50-60% of cases with congenital pulmonary airway malformation (CPAM), accessory spleen, congenital heart disease and congenital diaphragmatic hernia [1]. No chromosomal abnormalities have been associated with pulmonary sequestration [2, 4].

With advances in both fetal ultrasonography and magnetic resonance imaging, abnormalities of the thorax are increasingly being recognized antenatally, allowing providers to anticipate management issues at the time of delivery or later in neonatal life, and help parents comprehend the prognosis. Recognizing the imaging features of a variety of intrathoracic processes in the fetus is necessary for appropriate guidance of the clinicians caring for the mother [1, 3]. Ultrasonography is important in the diagnosis of pulmonary sequestration and its complications, in assessing progression, and in forming a prognosis, which, in turn, is important for appropriate parental counseling and fetal therapy. This imaging modality is noninvasive and safe, which make its use ideal in the prenatal and postnatal periods. In our case, the basal location of the lesions provides an excellent acoustic window for ultrasonography. The diagnosis can be made as soon as the early second trimester, as found in our case [2–5]. Color flow and duplex Doppler ultrasonography can elegantly depict the ectopic blood supply and drainage. In this case, the feeding artery originating from the descending aorta has seen at color Doppler US. Occasionally, these vessels may not be identified at Doppler US, making extralobar sequestration indistinguishable from a microcystic CPAM. Large lesions can compress the esophagus and thoracic veins and subsequently cause hydrops, which is an indication for fetal intervention or early delivery [1, 5]. Serial prenatal sonograms are necessary in patients in whom pulmonary sequestration is suspected, in order to search for poor prognostic factors, such as increasing mediastinal shift and increasing size of the sequestration.

In fetuses with chest masses, 8% have additional structural abnormalities and an abnormal karyotype. In patients with ELS, complications can include tension hydrothorax, polyhydramnios, and hydrops fetalis [2–4]. Ultrasonography can demonstrate absent or reversed diastolic flow in a torsed vascular pedicle, which is believed to cause complications in patients with ELS. The follow up during pregnancy is made by repeated US examinations to detect compressive pleural effusion wich may also induce fetal hydrops by cardiac and venous caval compression [3, 4, 5].

MR imaging and MRA can provide information without the need for ionizing radiation; however, MRI is less accessible, takes longer to perform, is subject to motion artifacts, and requires sedation in infants and small children [1, 6]. MR imaging may help in the diagnosis of pulmonary sequestration by demonstrating a solid, well-defined, uniformly hyperintense mass on T2-weighted images, and the feeding artery may be identified. Systemic blood supply is seen particularly arising from the aorta to a basal lung mass. In addition, MRA may demonstrate venous drainage of the mass and may obviate more invasive investigations. As in any imaging technique, MRI findings must be interpreted in the light of the clinical presentation and the ultrasonographic and chest radiographic findings [1, 5].

After birth, a chest X-ray finding of an elongated or cystic lesion, adjacent or posterior to the cardiac silhouette and described as a triangular, well-defined mass, is suggestive of the diagnosis [2]. A CT scan of the chest reveals the sequestered lung tissue and its vascularization, as well as other associated malformations. If present, The test of choice for the postnatal diagnosis is CT angiography. Until recently, conventional angiography was the gold standard for demonstrating the arterial supply and venous drainage of such lesions. Currently, CT angiography is used instead of conventional radiography because the former is less invasive and more efficient in diagnosing such lesions, showing the vascularization of the sequestered tissue in great detail and therefore allowing safer surgical planning [1, 2].

Extralobar pulmonary sequestration is macroscopically characterized by an oval or pyramidal lesion of 0.5-1.5 cm that can be larger in cases of recurrent infections. Histologically extralobar pulmonary sequestration is composed of irregular bronchi and bronchioles, as well as by alveoli that are twice to five times their normal size. Dilated lymphatic vessels in the subpleural region are found in 85% of cases [1, 2].

In the retroperitoneal location, mimics of ELS include neuroblastoma, adrenal hemorrhage, teratoma, and lymphangioma [6, 7]. A neuroblastoma is characterized by poorly defined margins and low or mixed echogenicity with foci of calcification. A neuroblastoma is more often cystic, right sided, and seen in the third trimester; ELS is more often echogenic and left sided and can possibly seen as early as the second trimester. Bronchogenic cyst, persistent pneumonia, pulmonary arteriovenous malformation, scimitar syndrome, Teratomas and lymphangiomas occur considerably less frequently [1, 2].

Patients who are asymptomatic at birth can subsequently develop cough, hemoptysis, and recurrent pneumonia, or can remain asymptomatic and be diagnosed incidentally (10% of extralobar sequestrations). Other patients can develop complications, such as hemoptysis, massive hemothorax, cardiovascular complications, fungal/bacterial infections, benign tumors, or malignant degeneration [3, 4] Symptomatic infants require prompt surgical resection, and the treatment of is sequestrectomy or pulmonary lobectomy at the time of diagnosis principally because of the possibility of recurrent infections, hemorrhage, malignant transformation, and other complications [1, 2, 4]. However, there is still controversy as to when resection should be performed. Many authors recommend that asymptomatic patients with extralobar sequestration remain under observation only, because such lesions rarely cause symptoms and because there have been no reports of malignant degeneration. However, in such lesions, a mixed component, with a morphology similar to that of CCAM, cannot be ruled out [2]. The surgical treatment of choice in cases of extralobar pulmonary sequestration is sequestrectomy in which the pedicle is carefully resected and ligated; sequestrectomy can also be performed through video-assisted thoracoscopy, a procedure that has been shown to be safe and to have a low rate of postoperative complications [1, 2, 4]. Coil embolisation has also been successfully trialled in selected cases [1]. Spontanenous involution has been reported in occasional cases [1, 5].

## Conclusion

Pulmonary sequestration refers to the situation whereby a portion of lung tissue receives its blood supply from an anomalous systemic artery. Pulmonary sequestrations are the second commonest congenital lung anomaly. The number of cases that are diagnosed early has increased thanks to new diagnostic techniques, such as CT, prenatal ultrasound, CT angiography, and nuclear magnetic resonance imaging.
